# Hypercaloric diet modulates hippocampal electrophysiological responses in a rat model of Alzheimer's Disease

**DOI:** 10.1002/alz70855_107129

**Published:** 2025-12-25

**Authors:** Andrea Trevisiol, Dustin Loren V Almanza, Margaret M Koletar, Aaron Y Lai, Mary Hill, Greg Stanisz, Joanne Mclaurin, Bojana Stefanovic

**Affiliations:** ^1^ Sunnybrook Health Sciences Centre, Toronto, ON, Canada; ^2^ Sunnybrook Research Institute, Toronto, ON, Canada; ^3^ University of Toronto, Toronto, ON, Canada; ^4^ Physical Sciences, Sunnybrook Research Institute, Toronto, ON, Canada; ^5^ Medical Biophysics, University of Toronto, Toronto, ON, Canada

## Abstract

**Background:**

Alzheimer's disease (AD) is characterized by impaired glucose metabolism, leading to neuronal energy deficits and disrupted neuronal network activity. Interventions aimed at normalizing metabolism are thus garnering increased research interest. Given that obesity is a known comorbidity of AD, we investigated how exposure to a high‐calorie, high‐fat (HCHF) diet affects neuronal function in the established stage of AD pathology in the TgF344‐AD rats.

**Method:**

TgF344‐AD (TgAD) and wild‐type (nTg) rats were fed either a standard chow diet; or a standard chow with a rotating set of HCHF diet items. After 3 months on the diet, neuronal activity in the somatosensory cortex and hippocampus was simultaneously recorded using Neuropixels electrodes. Local field potentials (LFPs) and spiking activity were analyzed at rest and in response to a 3Hz electrical stimulation of the forepaw. We reported stimulus‐evoked changes in spectral power, phase‐amplitude coupling (modulation index, MI), and spiking rate to evaluate how the HCHF diet modulates neuronal activity and network dynamics in the presence/absence of the transgenes.

**Result:**

LFPs of chow‐fed TgAD rats exhibited reduced resting alpha power and attenuated stimulation‐induced power increases (in delta, theta, and alpha bands) compared to nTg rats. HCHF feeding restored resting theta and alpha power in TgAD rats and enhanced stimulus‐induced power changes across delta, theta, and alpha bands. In the hippocampus, exposure to HCHF increased baseline theta and alpha power and potentiated MI (delta‐gamma, theta‐gamma) in TgAD rats. in response to forepaw stimulation in nTg rats. In response to forepaw stimulation, chow‐fed TgAD rats showed exaggerated MI changes compared to chow‐fed nTg rats, while HCHF feeding attenuated stimulation‐induced MI changes in both cohorts. While the HCHF diet attenuated spiking responses to forepaw stimulation in AD‐affected hippocampus, it had no effect in the cortex, where TgAD rats exhibited an overall lower response in their spiking activity.

**Conclusion:**

The HCHF diet partially rescues AD‐related deficits in neuronal activity by restoring baseline power and synchronization, suggesting that the HCHF diet may serve as a metabolic intervention by transiently normalizing neuronal activity in the metabolically‐challenged AD brain while being detrimental in normal aging.

